# Identification of two neutralizing human single-chain variable fragment antibodies targeting *Staphylococcus aureus* alpha-hemolysin 

**DOI:** 10.22038/IJBMS.2022.64103.14253

**Published:** 2022-10

**Authors:** Somayeh Piri-Gavgani, Mostafa Ghanei, Abolfazl Fateh, Seyed Davar Siadat, Leila Nematollahi, Fatemeh Rahimi-Jamnani

**Affiliations:** 1 Department of Mycobacteriology and Pulmonary Research, Pasteur Institute of Iran, Tehran, Iran; 2 Microbiology Research Center, Pasteur Institute of Iran, Tehran, Iran; 3 Chemical Injuries Research Center, Baqiyatallah University of Medical Sciences, Tehran, Iran; 4 Biotechnology Research Center, Pasteur Institute of Iran, Tehran, Iran

**Keywords:** Hemolysins, Monoclonal antibody, Single-chain variable – fragment, Staphylococcal infections, Staphylococcus aureus

## Abstract

**Objective(s):**

The inability of the host immune system to defeat *Staphylococcus aureus *is due to various secreted virulent factors such as leukocidins, superantigens, and hemolysins, which interrupt the function of immune components. Alpha-hemolysin is one of the most studied cytolysins due to its pronounced effect on developing staphylococcal infections. Alpha-hemolysin-neutralizing antibodies are among the best candidates for blocking the toxin activity and preventing *S. aureus* pathogenesis.

**Materials and Methods:**

A human single-chain variable fragment (scFv) phage display library was biopanned against alpha-hemolysin. The selected phage clones were assessed based on their binding ability to alpha-hemolysin. The binding specificity and affinity of two scFvs (designated SP192 and SP220) to alpha-hemolysin were determined by enzyme-linked immunosorbent assay. Furthermore, the neutralizing activity of SP192 and SP220 was examined by concurrent incubation of rabbit red blood cells (RBCs) with alpha-hemolysin and scFvs.

**Results:**

SP192 and SP220 showed significant binding to alpha-hemolysin compared with the control proteins, including bovine serum albumin, human adiponectin, and toxic shock syndrome toxin-1. Besides, both scFvs showed high-affinity binding to alpha-hemolysin in the nanomolar range (K*aff*: 0.9 and 0.7 nM^-1^, respectively), leading to marked inhibition of alpha-hemolysin-mediated lysis of rabbit RBCs (73% and 84% inhibition; respectively).

**Conclusion:**

SP192 and SP220 scFvs can potentially be used as alpha-hemolysin-neutralizing agents in conjunction with conventional antibiotics to combat S. aureus infections.

## Introduction

*Staphylococcus aureus *infections have become increasingly challenging due to the emergence of resistant strains and the low rate of development of new antibiotics (1). Life-threatening infections, such as endocarditis, pneumonia, and bacteremia with a high morbidity and mortality rate in patients with a suppressed immune system, infants, and people with diabetes, have led the National Academy of Science’s Institute of Medicine to rank methicillin-resistant *S. aureus *(MRSA) among the top 25 national priorities for research funding (2). Cytotoxins, including leukocidins (e.g., pantheon-valentine leukocidin), hemolysins (e.g., alpha-hemolysin), and phenol-soluble modulins (e.g., PSMα3), are the main invasiveness factors that directly impact the *S. aureus* pathogenesis (3-6). Alpha-hemolysin (so-called alpha-toxin) involves in the development and severity of* S. aureus* infections through attacking immune cells (e.g., human lymphocytes and monocytes), induction of apoptosis (e.g., endothelial cells), and disruption of endothelial and epithelial barrier integrity, resulting in dissemination of bacteria to bloodstream and other organs (3, 5, 7-10). It has been demonstrated that alpha-hemolysin plays a critical role in *S. aureus* infections such as brain abscess, dermonecrosis, pneumonia, and sepsis (5, 7, 11-13), making it an attractive target for the development of biotherapeutics.

Monoclonal antibodies (mAbs) have long been considered some of the most promising agents for neutralizing toxins, especially given their excellent safety profile and significant therapeutic efficacy (14-17). While the function of the constant fragment (Fc) region is not required for neutralizing the toxin and the binding of an antibody to the particular site of toxin is enough to block the activity of alpha-hemolysin, high-affinity and highly specific antibody fragments, such as fragment antigen-binding (Fab) and single-chain variable fragment (scFv), can be better alternatives than the full-length mAbs (18, 19). Caballero *et al*. developed a fully human anti-alpha-hemolysin Fab, LTM14, which could decrease corneal damage in rabbits with *S. aureus* keratitis (19). In contrast to Fab fragments, scFvs benefit from small size, easy production in bacteria, lower immunogenicity, and higher tissue penetration, making them potentially effective anti-toxin agents (20-23).

The current study aimed to identify alpha-hemolysin-specific scFv antibodies by screening a fully human scFv phage library on the alpha-hemolysin protein. Two scFvs (designated SP192 and SP220) were selected, and their binding characteristics and neutralizing ability were assessed *in vitro*.

## Materials and Methods

***Screening of a large human scFv phage library***

*Isolation of the scFv-phages specific to alpha-hemolysin*

To isolate phages expressing scFvs specific to alpha-hemolysin, a human scFv phage display library (diversity: 2×10^10^) was biopanned against the full-length alpha-hemolysin protein (Merck, Calbiochem, Germany) for four rounds as previously described (1, 24). A MaxiSorp 96-well microtiter plate (Nunc, Roskilde, Denmark) was coated with 100 µl of 2 μg/ml alpha-hemolysin in bicarbonate buffer 0.1 M or 100 μl of 4 μg/ml bovine serum albumin (BSA) (Merck) in phosphate-buffered saline (PBS). After incubation at 4 °C overnight, the plate was washed with PBS containing 0.05% (v/v) Tween-20 (PBS-T) and then incubated with blocking buffer (5 mg/ml BSA in PBS-T) for 90 min at room temperature (RT). Next, 100 µl of the scFv-phages (approximately 10^12 ^plaque-forming units [PFU]/ml) obtained from the library amplification (input_1_) were added to the BSA-coated wells, and incubation was done for one hour at RT. After 10 times washing with PBS-T, the bound phages were eluted by 10 min incubation with 150 µl of 0.2 M glycine-HCl (pH 2.2), followed by immediate neutralization with 1 M Tris-HCl (pH 9.1). The eluted phages (output_1_) were amplified in Escherichia coli strain TG1 and subjected to the next round of biopanning. The washing steps were repeated 10, 15, 20, and 25 times for rounds one to four to isolate the scFv-phages with high-affinity binding ability to alpha-hemolysin. 

*Assessment of the binding ability of the scFv-phages to alpha-hemolysin*

The binding ability of the phage pools obtained from four rounds of biopanning (input_1-_input_4_ and output_1_-output_4_) to alpha-hemolysin was assessed by polyclonal phage enzyme-linked immunosorbent assay (ELISA) (24). Briefly, a Maxisorp 96-well microtiter plate (Nunc) was coated with 100 μl of alpha-hemolysin (2 μg/ml) and then blocked with a blocking buffer. The wells coated with 100 μl of 4 μg/ml BSA in PBS were used as the control. Next, pre-blocked phages (~10^12^ PFU/ml) were added to the wells, and the incubation was done at RT for one hour. The wells were washed five times with PBS-T and once with PBS, and horseradish peroxidase (HRP)-conjugated mouse anti-M13 antibody (1:2000 in blocking buffer) (Santa Cruz Biotechnology Inc, Heidelberg, Germany) was added to the wells, followed by the incubation at RT for one hour. The wells were washed, and 3,3′,5,5′-Tetramethylbenzidine (TMB) (Thermo Scientific, MA, US) was added. The reactions were terminated by adding 1 M sulfuric acid (H_2_SO_4_) solution (Merck), and the optical density at 450 nm (OD_450_) was determined with an ELISA reader (Biotek, VT, USA). Next, the phages obtained from the last two rounds of biopanning, displaying the highest binding reactivity to alpha-hemolysin, were used to infect exponentially growing *E. coli* strain TG1 (24). More than 400 colonies were randomly picked and cultured in 2xTY medium containing ampicillin (100 µg/ml; Sigma, Steinheim, Germany) at 37 °C overnight. The binding ability of amplified phages to alpha-hemolysin was appraised by monoclonal phage ELISA (24).

***Expression of five soluble scFv antibodies***

Monoclonal soluble scFv antibodies were produced by infecting *E. coli *strain HB2151 with the five selected phages (clones SP164, SP178, SP192, SP218, and SP220), which showed the highest binding reactivity to alpha-hemolysin in polyclonal phage ELISA, as previously described by Soltanmohammadi *et al*. (1). The periplasmic expression of the scFvs was analyzed by a 12% sodium dodecyl sulfate-polyacrylamide gel electrophoresis (SDS-PAGE). Moreover, the expression of the scFvs was confirmed by western blotting as described previously (1). In brief, the periplasmic extract was run on a 12% SDS-PAGE gel and then transferred onto the polyvinylidene fluoride (PVDF) membrane (GE Healthcare) using a wet-tank transfer system. Next, the membrane was blocked with 5% (w/v) non-fat dry milk (Merck) in PBS. The membrane was incubated with the mouse polyclonal antibody generated against fully human scFvs (MAb) (1:200 dilution), followed by the goat anti-mouse mAb conjugated to HRP (GAb-HRP) (Santa Cruz) (1:2000 dilution). The bands were visualized using diaminobenzidine (DAB) (Sigma) and hydrogen peroxidase solution (H_2_O_2_) (Sigma). 

***Sequencing***

To analyze the sequences of five scFv antibodies, the phagemid DNA extraction was performed by the High Pure Plasmid Isolation Kit (Roche Diagnostics GmbH, Mannheim, Germany) as recommended by the manufacturer. A forward primer (5›-CTATGACCATGATTACGAATTTCTA-3›) was used to identify the nucleotide sequence of SP164, SP178, SP192, SP218, and SP220. Next, the sequences were analyzed in the IMGT/V-QUEST database (1). The data indicated that three scFvs, SP178, SP192, and SP218, had a similar sequence, and two scFvs, SP164 and SP220, shared a similar sequence. SP192 and SP220 were selected for further evaluation due to their higher expression levels.

***Investigation of the binding ability of two soluble scFvs to alpha-hemolysin***

The periplasmic extract containing the scFv antibody was purified by immobilized metal affinity chromatography (IMAC; Qiagen, Hilden, Germany), according to the manufacturer’s instructions. Next, the purified scFv antibodies were dialyzed against PBS in a dialysis bag with a molecular weight cut-off of 14 000 Da (Sigma), according to the manufacturer’s instructions. The concentration of the dialyzed scFvs was determined by the Bradford assay, and the purity of the scFvs was examined by SDS-PAGE. 

The binding of two soluble scFvs (SP192 and SP220) to alpha-hemolysin was examined by ELISA as previously described (24). In brief, a Maxisorp 96-well microtiter plate (Nunc) was coated with 100 μl of 2 μg/ml alpha-hemolysin protein or 4 μg/ml BSA. After blocking, 100 µl of the purified scFv (SP192 or SP220), the control scFv (MS460, an scFv specific to *S. aureus *TSST-1), or the mouse anti-staphylococcal alpha-hemolysin toxin mAb (mStaph-Alpha mAb) (6C12; IBT BIOSERVICES, Gaithersburg, MD, USA) (1:500 dilution) were added to the wells and incubation was done at RT for one hour. After several times washing, the wells were incubated with the MAb or normal mouse immunoglobin G (IgG) at RT for one hour. Next, the wells were washed, and GAb-HRP was added to the wells, followed by incubation at RT for one hour. After washing, the color reaction was developed with the TMB substrate solution and terminated by adding 1 M H_2_SO_4 _solution. OD_450_ was measured by an ELISA reader.

***Evaluation of binding specificity and affinity of two scFvs to alpha-hemolysin***

The specific binding and affinity of SP192 and SP220 to alpha-hemolysin were determined by ELISA (9, 24-26). To assess the binding specificity of SP192 and SP220, a Maxisorp 96-well microtiter plate (Nunc) was coated with 100 μl of alpha-hemolysin (2 μg/ml), human adiponectin (2 μg/ml) (R&D Systems, Minnesota, USA), BSA (4 μg/ml), non-fat dry milk (10 mg/ml), or TSST-1 (2 μg/ml) (Sigma). The binding of two scFvs to the coated proteins was detected with the MAb, followed by the GAb-HRP mentioned above. 

To measure the affinity of SP192 and SP220 to alpha-hemolysin, a MaxiSorp 96-well microtiter plate (Nunc) was coated with 100 µl of alpha-hemolysin (2 and 5 µg/ml). The alpha-hemolysin-coated wells were incubated with 100 µl of SP192 or SP220 at concentrations ranging from 0.02 to 450 μg/ml at RT for one hour. Next, the wells were incubated with MAb, followed by GAb-HRP. Using the following equation defined by Beatty *et al*. (27), the affinity constant (K_aff_) of SP192 and SP220 to alpha-hemolysin was calculated:

n = Ag / Ag′

K_aff_  = n - 1/2 (n [scFv′] - [scFv])

Where Ag and Ag’ are the concentrations of alpha-hemolysin (5 and 2 µg/ml, respectively), and scFv and scFv′ are the concentrations of SP192 (or SP220) at half maximum binding to alpha-hemolysin at concentrations of 5 and 2 µg/ml, respectively (OD50 and OD50’, respectively). 

***Determination of toxicity of alpha-hemolysin-specific scFvs***

*The toxic potential of two scFvs on rabbit red blood cells (RBCs)*

To investigate the hemolytic potential of SP192 and SP220, rabbit RBCs were treated with scFv as previously described (1, 28-30). Briefly, 100 µl of 5% (v/v) rabbit RBCs suspension in a round-bottom 96-well plate** (**Nunc**) **were incubated with SP192, SP220, or a combination of SP192 and SP220 (8.3 µM) at 37 °C for one hour. The wells containing rabbit RBCs incubated with normal saline (no hemoglobin release) or 0.1% (v/v) Triton X-100 (maximum hemoglobin release) were used as the controls. The microtiter plate was centrifuged, and the absorbance at 450 nm (A_450_) was measured to determine the quantity of hemoglobin released into the supernatants.

The hemolysis percentage was calculated with the following equation (1):

Hemolysis percentage = [A_450 scFv _- A_450 NS_] / [A_450 Triton X-100_ - A_450 NS_] × 100 

Where A_450 scFv _is the absorbance of the wells treated with SP192 or SP220, A_450 NS _is the absorbance of the wells treated with normal saline (NS), and A_450 Triton X-100 _is the absorbance of the wells treated with 0.1% Triton X-100. 

*Toxic potential of two scFvs on the human embryonic lung fibroblast cells (MRC-5)*

To examine the cytotoxic potential of SP192 and SP220, the MRC-5 cells (National Cell Bank, Pasteur Institute of Iran) were treated with SP192, SP220, or a combination of SP192 and SP220 (31). Briefly, in a flat-bottom 96-well cell culture plate (Nunc), the MRC-5 cells (10^4^ cells/well) in Dulbecco’s modified Eagle’s medium (Gibco, Grand Island, NY, USA) plus 10% fetal bovine serum (Gibco) were incubated with SP192 (6.6 µM), SP220 (6.6 µM), a combination of SP192 and SP220 (6.6 µM), alpha-hemolysin (0.15 µM), or PBS at 37 °C for 16 hr under 5% CO_2_. The toxic effects of SP192 and SP220 on the morphology of the MRC-5 cells were examined compared with the cytotoxic effect of alpha-hemolysin using an inverted microscope (BEL INV100, MA, Italy). 

***Evaluation of the neutralizing activity of the scFvs against alpha-hemolysin***

The effect of the binding of SP192 and SP220 to alpha-hemolysin on the hemolytic activity of the toxin was examined as previously described by Tkaczyk* et al*. (11) with some modifications. Briefly, in a round-bottom 96-well microtiter plate (Nunc), 100 µl of 5% (v/v) rabbit RBCs suspension were incubated simultaneously with alpha-hemolysin (5 nM), and a serial dilution of the scFv (SP192, SP220, and a combination of SP192 and SP220) (9.6 to 0.103 µM) at 37 °C for one hour. The wells containing rabbit RBCs incubated with I) normal saline, II) normal saline and alpha-hemolysin, or III) alpha-hemolysin and the EB211 scFv (an scFv against *Acinetobacter baumannii)* were used as the controls. Next, the reaction mixtures were centrifuged, and the absorbance of the released hemoglobin in the supernatant was measured at 450 nm. The inhibition percentage of alpha-hemolysin by the scFvs was calculated using the following equation (14):

Inhibition percentage = 100 - [100 × (*A*_450 Hla + scFv_) / (*A*_450 Hla_)]

*Where A*_450 Hla + scFv _is the absorbance of the wells treated with alpha-hemolysin (Hla) and scFv, and *A*_450 Hla _is the absorbance of the wells treated with alpha-hemolysin (Hla) alone*.*

***Statistical analyses***

The data were analyzed using the analysis of variance (ANOVA) or Student’s *t-*test where appropriate. GraphPad Prism v 6.0 was performed for statistical analyses. Probability values (*P*-values) of less than 0.05 were considered statistically significant.

## Results

***Identification of scFvs binding to alpha-hemolysin***

**Figure 1:**
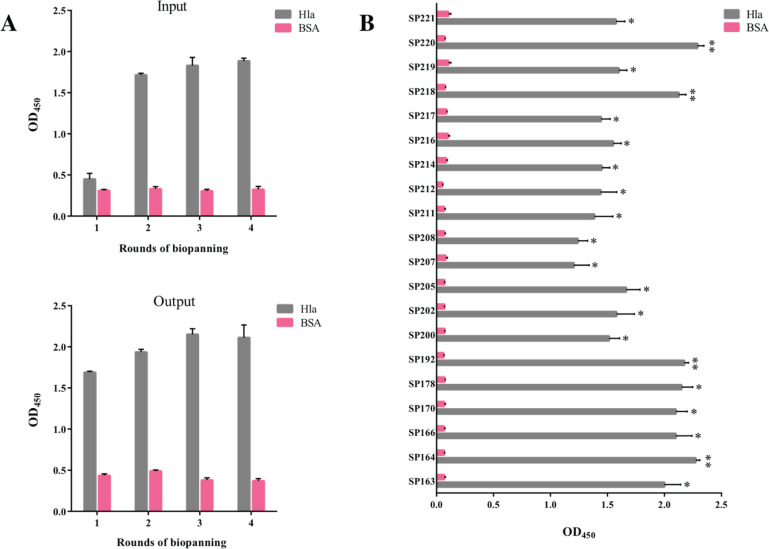
Assessment of the binding ability of phage clones to alpha-hemolysin by ELISA. (A) The binding reactivity of phage pools amplified from four rounds of biopanning on alpha-hemolysin (Hla) was analyzed by polyclonal phage ELISA. Bovine serum albumin (BSA)-coated wells were used as the control. (B) The binding of the 20 selected phage clones to alpha-hemolysin and BSA (control) was examined by monoclonal phage ELISA. Five phage clones (SP164, SP178, SP192, SP218, and SP220) exhibited higher binding abilities to alpha-hemolysin than BSA. The data are represented as the mean ± standard

Enriching a human scFv phage library with alpha-hemolysin led to isolating a population of scFv-phages. The polyclonal phage ELISA results showed that the scFv-phages obtained from the third and fourth rounds of biopanning reacted significantly with alpha-hemolysin compared with the control protein (BSA) (Figure 1A). To identify monoclonal phages specific to alpha-hemolysin, E. coli TG1 bacteria infected with the phages obtained from the last two rounds of biopanning were cultured on the lysogeny broth (LB) agar supplemented with ampicillin. An ELISA was used to assess the binding ability of phages amplified from single colonies to alpha-hemolysin. As shown in Figure 1B, out of 20 phage clones able to bind alpha-hemolysin, five clones, SP164, SP178, SP192, SP218, and SP220, showed the highest levels of binding. 

**Figure 2.**
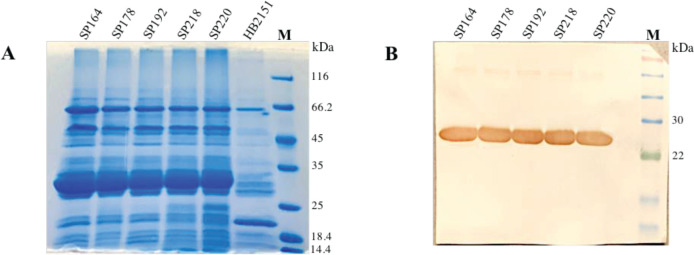
SDS-PAGE and western blot analysis of five soluble scFvs. (A) The periplasmic expression of five scFv antibodies, including SP164, SP178, SP192, SP218, and SP220, was analyzed by a 12% SDS-PAGE gel. Lane M: Unstained protein marker. (B) A sharp band corresponding to the scFv with a molecular weight of about 27 kDa was observed in western blot analysis. Non-infected *Escherichia* coli HB2151 was used as the control (HB2151). Lane M: Pre-stained protein marker

E. coli HB2151 bacteria were infected with the five selected phages to produce soluble scFvs (SP164, SP178, SP192, SP218, and SP220), which were subsequently evaluated by SDS-PAGE and western blot (Figure 2A and B). As illustrated in Figure 2B, a single band was observed at about 27 kDa, which corresponded to the molecular weight of the scFv (SP164, SP178, SP192, SP218, and SP220).

Based on the sequencing results, SP178, SP192, and SP218 shared the same sequence, and the sequence of SP164 and SP220 was identical. Therefore, SP192 and SP220 were selected for further characterization due to their high alpha-hemolysin binding ability and expression levels. Analysis of the nucleotide sequence by the IMGT/V-QUEST tool showed that the VH and VL of both scFvs belonged to human IGHV1-46*01F and IGKV1-39*01F germline genes, respectively. The amino acid sequence of both scFvs is presented in Supplementary Figure S1. 

***Significant binding of SP192 and SP220 to alpha-hemolysin***

**Figure 3:**
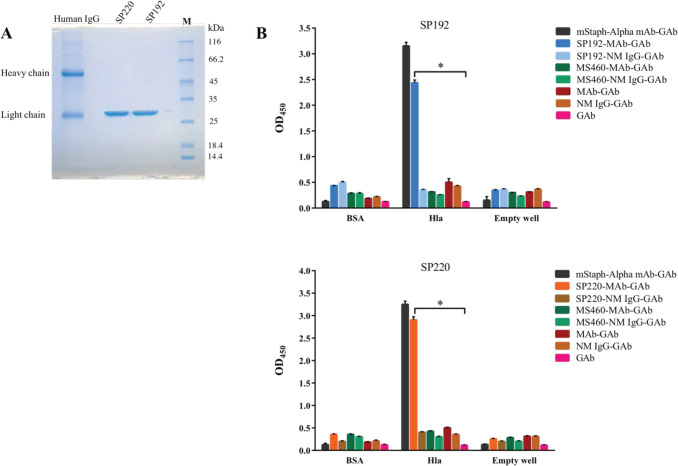
Binding ability of purified SP192 and SP220 scFvs to alpha-hemolysin. (A) SDS-PAGE. A single band corresponding to the scFv with a molecular weight of about 27 kDa was observed in a 12% SDS-PAGE gel. M: Unstained protein marker. (B) ELISA. The empty wells were used as the controls. Moreover, the wells incubated with the mouse anti-staphylococcal alpha-hemolysin toxin mAb (mStaph-Alpha mAb), followed by the goat anti-mouse mAb conjugated to HRP (GAb); SP192, SP220, or MS460, followed by normal mouse immunoglobin G (IgG) (NM IgG) and the GAb; MS460 (the control scFv), followed by the mouse polyclonal antibody generated against fully human scFvs (MAb) and the GAb; the MAb, followed by the GAb; NM IgG, followed by the GAb; or the GAb were used as the controls. The data are represented as the mean ± standard deviation of triplicate determination. Statistical significance was determined by the one-way analysis of variance (ANOVA) (* indicates *P*<0.05)

The amount of purified and dialyzed SP192 and SP220 was determined to be about 0.45 mg/ml. Based on SDS-PAGE results, a single band at about 27 kDa demonstrated the successful purification of SP192 and SP220 (Figure 3A). The binding of purified scFvs (SP192, SP220, and MS460 [as a negative control]) and mStaph-Alpha mAb (as a positive control) to alpha-hemolysin and BSA were investigated by ELISA. As shown in Figure 3B, SP192, SP220, and mStaph-Alpha mAb showed the highest binding to alpha-hemolysin compared with the controls.

***High specificity and binding affinity of SP192 and SP220 to alpha-hemolysin***

**Figure 4:**
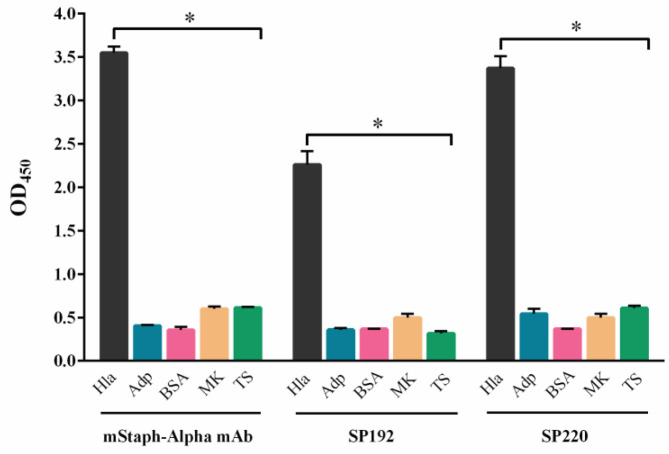
Binding specificity of SP192 and SP220. The specific binding of SP192 and SP220 to alpha-hemolysin was investigated by ELISA. SP192 and SP220 displayed significant binding to alpha-hemolysin, while minor cross-reactivity was observed with human adiponectin (Adp), bovine serum albumin (BSA), non-fat dry milk (MK), and toxic shock syndrome toxin-1 (TS). Mouse anti-staphylococcal alpha-hemolysin toxin mAb (mStaph-Alpha mAb) was used as a positive control. The data are represented as the mean ± standard deviation of triplicate determination. Statistical significance was determined by one-way analysis of variance (ANOVA) (* indicates *P*<0.05)

 The binding specificity of SP192 and SP220 was examined by ELISA. Based on the results, both scFvs displayed significant binding to alpha-hemolysin, while minor cross-reactivity was observed between the scFvs and BSA, human adiponectin, non-fat dry milk, and TSST-1 (Figure 4). 

***Negligible toxic effect of SP192 and SP220 on rabbit RBCs and MRC-5 cells***

**Figure 5:**
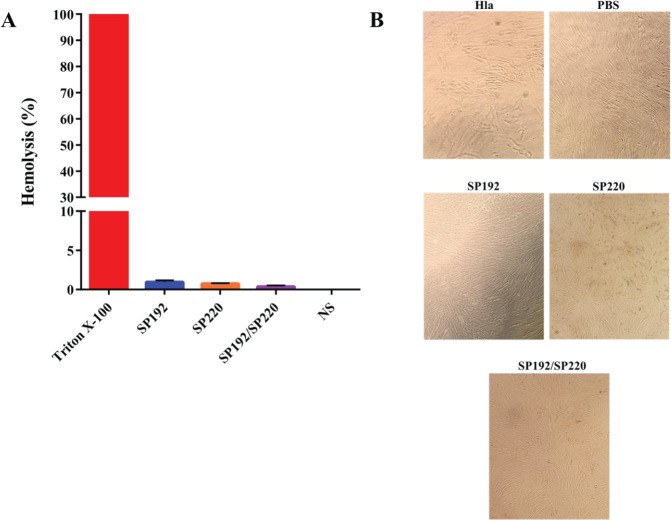
Assessment of the toxic effects of SP192 and SP220 on rabbit RBCs and MRC-5 cells. (A) Hemolytic activity of SP192 and SP220 on rabbit RBCs. SP192 and SP220 and a combination of SP192 and SP220 (SP192/SP220) showed no significant hemolysis (1.04%, 0.75%, and 0.38% hemolysis, respectively) compared with Triton X-100 (100% hemolysis). Rabbit RBCs incubated with normal saline (NS) were used as the control group. The data are represented as the mean ± standard deviation of triplicate determination. (B) Cytotoxic activity of SP192 and SP220 on the MRC-5 cells. SP192 and SP220 and a combination of SP192 and SP220 (SP192/SP220) had no toxic effects on the cells compared with alpha-hemolysin (Hla). The cells incubated with phosphate-buffered saline (PBS) were used as the control group

Treatment of rabbit RBCs with SP192, SP220, or a combination of SP192 and SP220 showed no significant hemolysis (1.04%, 0.75%, and 0.38% hemolysis, respectively) compared with rabbit RBCs treated with Triton X-100 (100% hemolysis) (Figure 5A). Furthermore, the cell morphology of the MRC-5 cells incubated with SP192 and SP220 (alone or a combination of two scFvs) was compared with the MRC-5 cells incubated with PBS (as a negative control). As illustrated in Figure 5B, no evident morphological changes were observed between the scFv- and PBS-treated groups. In contrast, alpha-hemolysin exhibited a significant toxic effect on the MRC-5 cells (Figure 5B).

***Significant inhibitory effect of SP192 and SP220 on the hemolytic activity of alpha-hemolysin***

**Figure 6:**
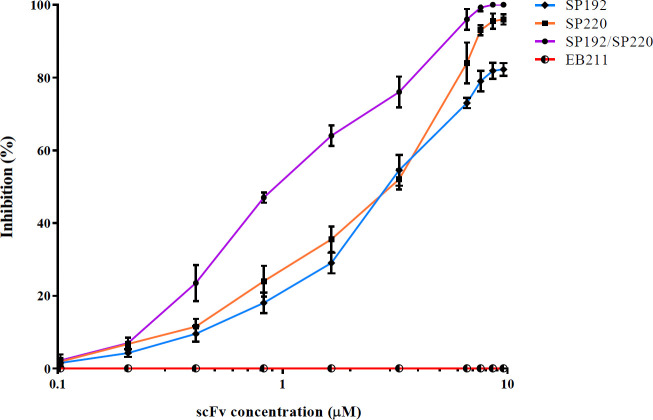
Inhibition of alpha-hemolysin-mediated lysis of rabbit RBCs by SP192 and SP220. SP192, SP220, and a combination of SP192 and SP220 (SP192/SP220) neutralized the hemolytic activity of alpha-hemolysin in a dose-dependent manner. In contrast, EB211 (the control scFv against Acinetobacter *baumannii*) could not inhibit the hemolytic activity of alpha-hemolysin

The inhibitory activity of SP192 and SP220 (alone and a combination of two scFvs) on the hemolytic effect of alpha-hemolysin was assessed by the concurrent treatment of rabbit RBCs with alpha-hemolysin and a range of concentrations of scFvs (SP192, SP220, a combination of SP192 and SP220, or EB211). As illustrated in Figure 6, SP192 showed an inhibition efficiency of 80% at concentrations of 8.6 and 9.6 µM compared with the control group (rabbit RBCs treated with normal saline and alpha-hemolysin). In contrast, SP220 exhibited more than 90% inhibitory activity at concentrations of 7.6–9.6 µM. Furthermore, a combination of two scFvs displayed a marked inhibition effect on the alpha-hemolysin-mediated RBCs lysis (64%, 76%, and 96% at concentrations of 1.65, 3.3, and 6.6, respectively, and 100% at concentrations of 7.6, 8.6, and 9.6 µM). Furthermore, EB211 had no impact on the hemolytic activity of alpha-hemolysin (Figure 6). 

deviation of triplicate determination. Significance was determined by Student’s *t*-test (* indicates *P*<0.01 and ** indicates *P*<0.001)

## Discussion

The binding of alpha-hemolysin to A-disintegrin and metalloprotease 10 (ADAM10) triggers a cascade of events, including the cell detachment from the neighboring cells and the basal membrane and cell lysis, dependent on the concentration of toxin and the expression of ADAM10 cell (8, 32-35). Therefore, direct targeting of alpha-hemolysin and ADAM10 can be functional strategies to prevent the detrimental effects of alpha-hemolysin (36). Notably, anti-alpha-hemolysin mAbs are one of the most promising neutralizing agents, exhibiting considerable activity in combating *S. aureus* infections (6, 9, 16, 37-39). Neutralization of alpha-hemolysin does not need the Fc-related activity of antibodies; therefore, antibody fragments such as scFvs are valuable substitutes due to their small size and high ability to penetrate the infected tissues, low immunogenicity, and easy and low-cost production compared with the full-length mAbs (18, 20, 21). To isolate anti-alpha hemolysin scFvs, we biopanned a fully human scFv phage library against alpha-hemolysin, leading to isolating five scFvs with high binding ability to the target toxin. Among isolated scFvs, two scFvs, SP192 and SP220, with unique sequences and high expression levels, were assessed based on their binding specificity and affinity to alpha-hemolysin and neutralization activity. Both scFvs showed significant binding to alpha-hemolysin, while no significant cross-binding was observed between the scFvs and proteins such as human adiponectin and TSST-1. Of note, SP192 and SP220 showed high affinity-binding to alpha-hemolysin (K_aff_ = 0.9 and 0.7 nM^-1^, respectively). Several studies reported the association between the binding affinity and the neutralization potency of anti-toxin antibodies (6, 11, 39-41). In this regard, we speculated that SP192 and SP220 showing high-affinity binding to alpha-hemolysin might have a significant neutralization activity against alpha-hemolysin. However, before evaluating the inhibitory activity of SP192 and SP220 on the hemolysis effect of alpha-hemolysin on rabbit RBCs, the toxic potential of the scFvs on rabbit RBCs and MRC-5 cells was assessed. Based on the results, neither scFvs had any hemolytic or cytotoxic activity. Next, the antagonist activity of SP192 and SP220 on alpha-hemolysin was examined by treating rabbit RBCs with alpha-hemolysin and different concentrations of SP192, SP220, and a combination of two scFvs. The results demonstrated that SP192 and SP220 significantly inhibited the lysis of rabbit RBCs compared with the control group. In two studies, Foletti *et al*. (6) and Caballero *et al*. (19) investigated the neutralizing activity of a human anti-alpha-hemolysin mAb (LTM14) and its Fab, respectively. They showed that LTM14 mAb (*K*_D_: 1.7 pM) and LTM14 Fab inhibited alpha-hemolysin-mediated lysis of rabbit RBCs (6, 19). In another study, Liu *et al*. developed a fully human mAb against alpha-hemolysin (YG1) with a *K*_D_ value of approximately 2 nM (9). Similar to the LTM14 mAb, the YG1 mAb inhibited the hemolytic activity of alpha-hemolysin in a dose-dependent manner (9). The LC-10 mAb, further named MEDI4893*, is a human IgG1 mAb, with a *K*_D _value of 0.6 nM, developed by Tkaczyk *et al*. (11). They showed that the LC-10 mAb impeded alpha-hemolysin-induced hemolysis in a dose-dependent fashion, and there was a relationship between the affinity and potency of anti-alpha hemolysin mAbs developed in this study (11). 

There is a long list of neutralizing scFvs developed against toxins such as adenylate cyclase toxin (*Bordetella pertussis*), anthrax toxin (*Bacillus anthracis*), botulinum neurotoxin (*Clostridium botulinum*), cry toxin (*Bacillus thuringiensis*), type A alpha-toxin (*Clostridium perfringens*), enterotoxin (*E. coli*), exotoxin A (*Pseudomonas aeruginosa*), hemolysin (*Vibrio parahaemolyticus*), Shiga toxin (*Enterohemorrhagic E. coli*), tetanus toxin (*Clostridium *tetani), and TSST-1 (*S. aureus*) (23). Most antibodies generated against alpha-hemolysin are conventional mAbs or bispecific antibodies such as 11H10-BiSAb comprising the scFv of MEDI4893* fused to the heavy chain of an anti-clumping factor A mAb (11H10) (6, 9, 11, 14). However, we demonstrated in the current study that a single scFv had the ability to neutralize alpha-hemolysin effectively.

Targeting various sites of the toxin with two or more neutralizing antibodies seems to be a sophisticated strategy for inhibiting the toxin-mediated cytotoxic effects. We showed that the combination of SP192 and SP220 (at a concentration of 6.6 µM) had higher inhibitory activity than SP192 and SP220 alone (96% versus 73% and 84%, respectively). Likewise, Demarest *et al*. reported that a cocktail of two neutralizing mAbs, designated 3358 and 3359, targeting *Clostridium difficile *toxin A, had higher neutralizing activity than each mAb alone (42). They suggested that binding two different mAbs to several epitopes on toxin A might result in efficient neutralization and subsequent decrease of toxin A-mediated cell lysis (42). 

## Conclusion

Alpha-hemolysin plays a critical role in the development of *S. aureus* infections. Furthermore, most *S. aureus* isolates express alpha-hemolysin, making it an excellent target for generating therapeutics effective against *S. aureus* infections. Our study led to the development of two novel human scFvs, SP192 and SP220, which bound significantly to alpha-hemolysin. Both scFv antibodies showed neutralization activity against alpha-hemolysin and significantly inhibited the lysis of rabbit RBCs mediated by alpha-hemolysin. It is therefore expected that the use of antibiotics in combination with two different anti-alpha hemolysin scFvs can lead to promising results in patients with *S. aureus* infections (e.g., pneumonia).

## Authors’ Contributions

FRJ Supervised, directed, and managed the study. MG, AF, SDS, and LN Helped design the study. SPG Performed the experiments and was involved in the manuscript preparation. All authors reviewed the manuscript. 

## Ethical Approval

The animal experiment was conducted in accordance with ARRIVE guidelines (https://arriveguidelines.org) and approved by the Animal Care and Use Committees of the Pasteur Institute of Iran (Ethics No.: IR.PII.REC.1398.031).

## Conflicts of Interest

The authors declare no competing interests. 
